# The association between hearing impairment and incident depression in older adults: a longitudinal analysis

**DOI:** 10.1093/gerona/glaf250

**Published:** 2025-11-07

**Authors:** Malcolm P Forbes, Mark W Cox, Katharine Brewster, Mojtaba Lotfaliany, Mohammadreza Mohebbi, Gary Rance, Robyn L Woods, Carlene Britt, John J Mcneil, Michael Berk

**Affiliations:** The Institute for Mental and Physical Health and Clinical Translation (IMPACT), School of Medicine, Deakin University, Geelong, Victoria, Australia; Barwon Health, Geelong, Victoria, Australia; Barwon Health, Geelong, Victoria, Australia; Department of Psychiatry, Columbia University Vagelos College of Physicians and Surgeons, New York, New York, United States; The Institute for Mental and Physical Health and Clinical Translation (IMPACT), School of Medicine, Deakin University, Geelong, Victoria, Australia; The Institute for Mental and Physical Health and Clinical Translation (IMPACT), School of Medicine, Deakin University, Geelong, Victoria, Australia; Faculty of Health, Biostatistics Unit, School of Medicine, Deakin University, Geelong, Victoria, Australia; Department of Audiology and Speech Pathology, The University of Melbourne, Melbourne, Victoria, Australia; School of Public Health and Preventive Medicine, Monash University, Melbourne, Victoria, Australia; School of Public Health and Preventive Medicine, Monash University, Melbourne, Victoria, Australia; School of Public Health and Preventive Medicine, Monash University, Melbourne, Victoria, Australia; The Institute for Mental and Physical Health and Clinical Translation (IMPACT), School of Medicine, Deakin University, Geelong, Victoria, Australia; Barwon Health, Geelong, Victoria, Australia

**Keywords:** Hearing loss, Older adults, Depression, Hearing aids, Audiometry

## Abstract

**Background:**

Hearing loss (HL) is common in older adults and is associated with several adverse health outcomes. Although previous research has demonstrated a link between hearing impairment and depression, most studies have been cross-sectional or relied on a single baseline measure of hearing. To investigate the association between longitudinal, time-varying audiometric measures of hearing and incident depression in older adults. A secondary aim was to assess whether hearing aid use modifies this association over time in those with moderate to severe HL.

**Methods:**

We included 1260 participants who underwent pure-tone audiometry at baseline, 18 months, and 36 months. Depression was defined using the CES-D-10 scale, with a cut-score of ≥8. Cox proportional hazards analyses were used to examine the link between hearing status (normal, mild HL, moderate/severe HL) and depression, adjusted for demographic, lifestyle, and clinical variables.

**Results:**

Over a median follow-up of 7.3 years, participants with moderate to severe HL had a higher risk of incident depression (adjusted HR [aHR]: 1.24; 95% CI, 1.08-1.43, *P* < 0.01) compared with participants with normal hearing. Hearing aid use (≥6 h self-reported use per day on average) in those with moderate to severe HL was associated with significantly reduced risk of incident depression (aHR: 0.65; 95% CI, 0.49-0.87, *P* < .01).

**Conclusion:**

Moderate to severe HL is a significant risk factor for incident depression among older adults. Hearing aid use attenuated this risk. Future research should investigate mechanistic pathways linking HL and mood disturbances.

## Introduction

Hearing loss (HL) affects more than 1.5 billion people worldwide, with more than 20% experiencing disabling hearing impairment.^1^ Although HL can be modified by amplification devices such as hearing aids, age-related HL typically progresses gradually, making it difficult to notice in the early stages.^2^ HL may lead older adults to withdraw from situations where they have difficulty hearing and communicating, contributing to isolation.^3^ In addition to barriers to social engagement, HL may threaten personal independence.^4^ Consequently, HL has been linked to a broad array of negative health outcomes, including reduced quality of life, cognitive impairment, and depression.^5^

A meta-analysis investigating the association between HL and depression in older adults revealed substantial study heterogeneity and mixed findings.^6^ Some studies have reported an increased risk of depression with HL,^2^^,^^7-18^ while others have reported no significant association.^5^^,^^19-27^ A limitation of most studies is the reliance on self-reported HL as the primary exposure measurement, rather than rigorous audiometric assessments. There is evidence that older adults with mild HL may be unaware of their impairment, leading to overestimation of their hearing abilities when self-reporting.^28^ Depressed older adults may be more likely to self-report HL, as late-life depression often presents in conjunction with somatic symptoms.^29^ Additionally, few longitudinal studies categorize HL by severity or consider the impact of hearing aid usage on depression.

There is a need for research in this area that addresses the methodological issues of the extant literature, given the prevalence and consequences of both age-related HL and depression. Evidence from regular hearing screening programs highlights the effectiveness of early detection and intervention, with auditory amplification devices improving listening ability and quality of life,^30-32^ as well as reducing dementia risk.^33^ Few studies have explored the dose–response relationship between audiometrically assessed HL and depression and whether hearing aid use can mitigate depressive symptoms. In response to this knowledge gap, this longitudinal study investigates the dose–response relationship between audiometrically assessed HL and depressive symptoms over several years and explores whether hearing aid use can mitigate these symptoms.

## Methods

### Study population

This study utilized data from the ASPREE (ASPirin in Reducing Events in the Elderly) trial and its extension, ASPREE-XT.^34^^,^^35^ ASPREE was an international, multicenter, randomized controlled trial designed to evaluate whether daily low-dose aspirin could prolong healthy, disability-free life in older adults by preventing age-related conditions, including cardiovascular disease, dementia, and depression. Conducted between 2010 and 2017, the trial enrolled 19 114 healthy participants aged 70 years or older (65 years for US minorities) who were free of dementia, independence-limiting physical disability, and known cardiovascular disease at the time of enrollment. Participants included 16 703 from Australia and 2411 from the United States. The ASPREE-XT extension study commenced in 2018, transitioning the cohort into a longitudinal observational study to further explore factors contributing to healthy aging, including cognitive and physical health outcomes. Participants were followed up face-to-face each year, and by telephone each 6 months. For this analysis, we focused on a subset of 1260 ASPREE participants with complete baseline data on hearing ability, depressive symptoms, and relevant covariates.^36^

### Measures and operational definitions

Hearing ability was measured at baseline, 18 months, and 36 months and was assessed using World Health Organisation (WHO) standards for pure-tone audiometry.^37^^,^^38^ Average tone sound detection thresholds for tones at 0.5, 1, 2, and 4 kHz test frequencies in the better ear were calculated, and ears were categorized as “normal” hearing (≤25 dBHL), “mild” HL (26-40 dBHL), or “moderate to severe” HL (≥41 dBHL). We additionally classified hearing aid use as present if participants reported wearing a hearing aid in at least 1 ear, or absent if otherwise. We further assessed hearing aid use as frequent (≥6 h hearing aid use per day) or infrequent (<6 h hearing aid use per day). Incident depression was defined as a Center for Epidemiologic Studies Depression Scale 10-item (CES-D-10) score of ≥8 during the follow-up period. A cut-score of 8 was chosen to improve sensitivity in detecting depression.^39^ The CES-D-10 has been shown to have reliability and validity with respect to a diagnosis of depression among older adults.^39^ Depressive symptoms were assessed annually, and participants were followed up for a median time of 7.3 years (IQR 0.21 years).

### Statistical analysis

Cox proportional hazards regression models were employed to evaluate the association between time-updating hearing ability (normal, mild, moderate/severe) and risk of developing depression (CES-D-10 ≥ 8). Hearing ability and hearing aid use were both treated as time-varying exposures, allowing participants to transition between hearing status categories or update hearing aid use across baseline, 18-month, and 3-year visits. Depression was analyzed as a recurrent event, meaning individuals who recovered (ie, had a CES-D-10 score below the threshold at subsequent visits) re-entered the risk pool, allowing additional depressive episodes to be analyzed. Start–stop intervals were created to capture these transitions over time in Cox models. In contrast, the Kaplan–Meier curves focused on time to the first depressive episode only, censoring participants at the earliest occurrence of depression onset, last known study contact, withdrawal, or death. For first-onset incidence models, the event was the first assessment with CES-D-10 ≥ 8 after baseline among those non-depressed at baseline (excluding those with CES-D-10 ≥ 8 or reporting antidepressant use at baseline). In recurrent-event models, we did not exclude those with baseline CES-D-10 ≥ 8. Covariates included in adjusted models were selected based on published evidence linking them to both HL and depression.^40^ These consisted of age, sex, living arrangement, educational attainment, smoking history, alcohol use, cognitive performance (modified Mini-Mental State score), body mass index, diabetes, and chronic kidney disease. These covariates were chosen based on a review of the literature, and further details on their measurement can be found in McNeil et al.^34^ The proportional hazards assumption was tested by examining Schoenfeld residuals, revealing no significant violations. Hazard ratios (HRs) with 95% CIs are presented, providing the time-averaged risk over the entire follow-up period, with normal hearing as the reference category. Missingness was low, with 90.5% of participants having complete data, and missingness was primarily driven by 1 variable. Missing data were handled using a complete-case approach, with participants with missing values on any exposure, outcome, or covariate excluded. All statistical analyses were conducted in R version 4.2.0.^41^

### Subgroup analysis

In addition to the main models, we performed a subgroup analysis restricted to participants who were classified as having moderate/severe hearing impairment at any time point. Among participants classified as having moderate/severe HL at any time point, we conducted 2 pre-specified comparisons with time-updated exposures: (1) any hearing-aid use versus no use (reference = no use); and (2) among hearing-aid users, ≥6 h/day versus <6 h/day (reference ≤6 h/day). The ≥6-h threshold was defined to approximate “most-of-day” use.

## Results

### Baseline characteristics

The baseline characteristics of the study participants, stratified by hearing category (normal, mild, and moderate/severe HL), are presented in Table 1. Participants with moderate/severe HL at baseline were more likely to be older and living alone. Hearing aid use was reported in 2.2% of those with no pure tone audiometry impairment, 21.5% of those categorized as having mild HL, and 73.5% of those with moderate to severe HL. Baseline HHIE-S, with higher scores indicating a greater perceived hearing handicap,^42^ was correlated with baseline audiometry-assessed HL (Pearson’s *r* = 0.62 [95% CI, 0.61-0.64, *P* < .01]).

**Table 1.** Baseline characteristics of the study population.

	**Normal** (*n* = 634)	**Mild** (*n* = 437)	**Moderate/severe** (*n* = 189)	**Total** (*n* = 1260)
**Age, mean (SD)**	73.4 (3.17)	74.9 (4.45)	76.5 (4.94)	74.4 (4.10)
**Sex, *n* (%)**				
** Male**	257 (40.5)	248 (56.8)	98 (51.9)	603 (47.9)
** Female**	377 (59.5)	189 (43.2)	91 (48.1)	657 (52.1)
**Living status, *n* (%)**				
** Living alone**	178 (28.1)	122 (27.9)	70 (37.0)	370 (29.4)
** Living with others**	454 (71.6)	313 (71.6)	119 (63.0)	886 (70.3)
**Smoking history, *n* (%)**				
** Current**	16 (2.5)	20 (4.6)	4 (2.1)	40 (3.2)
** Former**	241 (38.0)	199 (45.5)	78 (41.3)	518 (41.1)
** Never**	377 (59.5)	218 (49.9)	107 (56.6)	702 (55.7)
**Alcohol use, *n* (%)**				
** Current**	521 (82.2)	364 (83.3)	149 (78.8)	1034 (82.1)
** Former**	31 (4.9)	15 (3.4)	13 (6.9)	59 (4.7)
** Never**	82 (12.9)	58 (13.3)	27 (14.3)	167 (13.3)
**Education level ≥12 years, *n* (%)**	333 (52.5)	254 (58.1)	121 (64.0)	708 (56.2)
**3MS score (mean [SD])^a^**	94.1 (4.34)	93.0 (4.37)	92.7 (4.21)	93.5 (4.36)
**Baseline depression score (CES-D-10), mean (SD)**	3.46 (3.41)	3.57 (3.76)	3.51 (3.08)	3.51 (3.49)
**Baseline CES-D-10 score ≥8**	70 (11%)	52 (11.9%)	24 (12.7%)	146 (11.6%)
**Diabetes, *n* (%)^b^**	74 (11.7)	54 (12.4)	20 (10.6)	148 (11.7)
**Chronic kidney disease (CKD), *n* (%)^c^**	107 (16.9)	97 (22.2)	37 (19.6)	241 (19.1)
**Hearing aid use, *n* (%)**	14 (2.2)	94 (21.5)	139 (73.5)	247 (19.6)

Abbreviation: CES-D-10, Center for Epidemiologic Studies Depression Scale 10-item.

a 3MS is the modified mini-mental state examination.

b Defined as self-report or the use of any drug use for the treatment of diabetes, including insulin, or a fasting blood glucose level of greater than or equal to 7 mmol/L.

c Defined an estimated glomerular filtration rate of less than 60 mL/min/1.73m^2^.

### Depression-free survival

**Figure 1.**
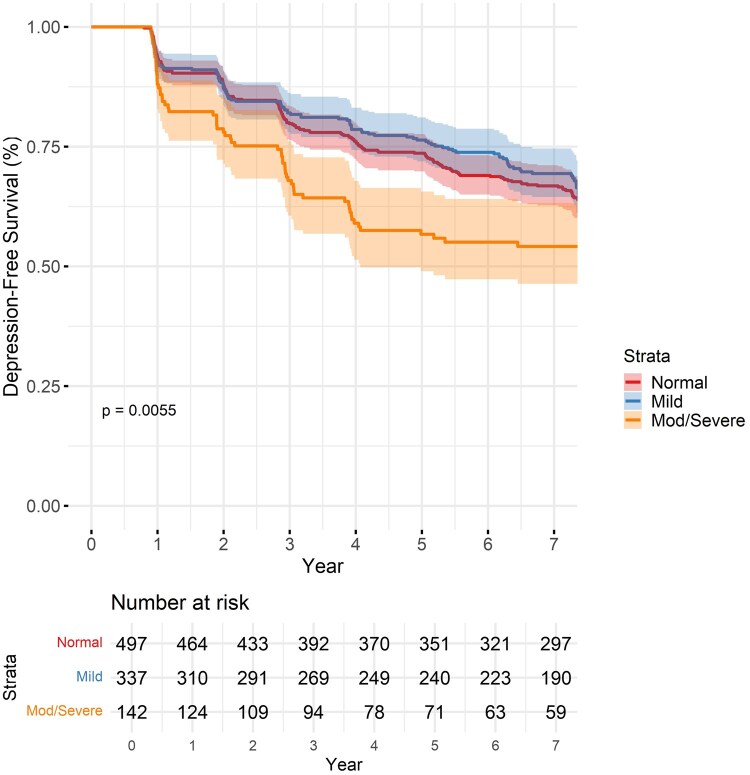
Kaplan–Meier curve of baseline hearing category and depression incidence.

The Kaplan–Meier curve demonstrated depression-free survival over time, stratified by hearing impairment categories (Figure 1). It highlighted differences in survival trajectories, with individuals with moderate/severe HL exhibiting lower probabilities of remaining depression-free compared to those with mild or normal hearing (χ^2^(2) = 8.8, *P* = .013). In Cox proportional hazards models, moderate to severe HL was associated with an increased risk of incident depression over the follow-up period. In the fully adjusted model (Table 2), there was an adjusted HR (aHR) of 1.24 (95% CI, 1.08-1.43, *P* < .01). Mild HL was not significantly associated with depression risk in unadjusted or adjusted models. Complete case analysis was utilized; however, the impact of missing data was evaluated using multiple imputation, and there were no differences in the findings.

**Table 2.** Cox proportional hazards model for risk of depression based on hearing impairment.

	Normal hearing	Mild hearing loss	Moderate-severe hearing loss
**IR per 100 PY (95% CI)^a^**	19.3 (17.8-20.8)	19.3 (17.8-20.8)	23.9 (21.5-26.5)
**Model 1 (95% CI)^b^**	1.00	1.01 (0.90-1.13)	1.24 (1.08-1.42)
**Model 2 (95% CI)^c^**	1.00	1.04 (0.92-1.16)	1.25 (1.09-1.45)
**Model 3 (95% CI)^d^**	1.00	1.03 (0.92-1.16)	1.24 (1.08-1.43)

Abbreviations: IR, incidence rate; PY, per year.

a Incidence rate per 100 person-years.

b Model 1 adjusted for age and sex.

c Model 2 adjusted for age, sex, living status, smoking history, and alcohol use.

d Model 3 adjusted for age, sex, living status, smoking history, alcohol use, body mass index, cognition, and the presence of diabetes and chronic kidney disease.

In a subgroup analysis restricted to the moderate to severe HL subgroup, any self-reported use versus no use of hearing aids was not associated with incident depression (aHR = 1.03, 95% CI, 0.80-1.32) (Table 3). However, among hearing aid users, more than 6 h of self-reported use per day was associated with lower risk compared with less than 6 h of use per day (aHR = 0.65, 95% CI, 0.49-0.87) (Table 4). Modeling hours of use per day continuously showed a dose–response (aHR per hour = 0.98; 95% CI, 0.97-0.99).

**Table 3.** Cox proportional hazards model for hearing aid use and risk of depression in subgroup with moderate to severe hearing loss.

	No hearing aid use	Hearing aid use
**IR per 100 PY (95% CI)**	23.8 (19.1-29.3)	24.1 (21.2-27.4)
**Hazard ratio (95% CI)^a^**	1.00	1.03 (0.81-1.32)

Abbreviations: IR, incidence rate ; PY, per year.

a Model adjusted for age, sex, living status, smoking history, alcohol use, body mass index, cognition, and the presence of diabetes and chronic kidney disease.

**Table 4.** Cox proportional hazards model for ≥ 6 versus < 6 hours of hearing aid use in subgroup with moderate to severe hearing loss.

	< 6 h	≥6 h
**IR per 100 PY (95% CI)**	34.0 (26.4-43.1)	22.0 (19.0-25.5)
**Hazard ratio (95% CI)**	1.00	0.65 (0.49-0.87)

Abbreviations: IR, incidence rate ; PY, per year.

Model adjusted for age, sex, living status, smoking history, alcohol use, body mass index, cognition, and the presence of diabetes and chronic kidney disease.

## Discussion

This prospective cohort study of 1260 older adults found that moderate to severe HL, measured using pure tone audiometry, was associated with an increased risk of incident depression after adjusting for a range of potential confounders. In the fully adjusted model, moderate/severe HL was associated with a 1.24-fold increase in depression risk over a period averaging 7.3 years. In contrast, mild HL was not significantly associated with depression risk. Notably, hearing aid use for more than 6 h per day exerted a protective effect, suggesting that consistent use of auditory amplification may mitigate the psychological consequences of HL.

This study adds to the growing body of evidence linking HL to depression in older adults. Importantly, our study is one of the few to use robust audiometric measures of hearing ability in a longitudinal cohort. These measures psychophysically determine sound detection levels, and as such, are less prone to biases associated with self-reported data.^43^ Previous studies, reported in recent meta-analyses,^6^^,^^40^^,^^44^ have largely relied on self-reported HL, which may underestimate the true prevalence of HL and its impact on mental health and may be affected by the presence of depression. By using pure tone audiometry in a longitudinal study design, our study provides the most reliable assessment of HL severity and its relationship with subsequent depression.

Our findings are consistent with prior research showing a modest association between HL and depression. Notably, the effect found in our study, which was longitudinal in nature and accounted for a range of potential confounders, was lower than previous meta-analytic estimates.^6^ This may relate to the sample, which was older adults eligible for a primary prevention trial. Nonetheless, our results align with research that identifies a stronger association with depression for more severe degrees of HL. For example, the findings of Powell et al.^7^ in the Health ABC Study, which similarly showed that older adults with moderate or greater HL had an approximately 25% higher risk of developing clinically significant depressive symptoms compared to those with normal hearing. Like Powell et al., we observed that mild HL was not consistently linked to depression risk, underscoring that the greatest vulnerability may lie among those with more pronounced impairment.

There have been mixed findings about the effects of hearing aid use on depression risk. Our study is unique in measuring the effect of self-reporting hours of hearing aid use, rather than self-reported hearing aid use as a binary outcome. Consistent with previous studies,^7^^,^^8^^,^^12^^,^^18^ our study found that self-reported hearing aid use as a yes/no response was not associated with a reduced risk of depression. However, when measured as self-reported hearing aid use for more than 6 h per day, hearing aid use was found to be protective (HR = 0.65, *P* = .003) in individuals with moderate to severe HL. Our finding is supported by Tsimpida et al.^9^ who examined longitudinal data from the English Longitudinal Study of Ageing (ELSA) and found that older adults with hearing impairment who used hearing aids experienced fewer depressive symptoms than non-users, suggesting a moderating effect of hearing aid use on the relationship between HL and depression. These benefits were more pronounced among participants with greater social or economic vulnerabilities, indicating that consistent hearing aid use may be especially protective in groups at higher risk for late-life depression. Adherence to hearing aid use is generally suboptimal, with 55.4% of people prescribed a hearing aid using them daily and only 27.3% using them >6 h a day, in 1 study.^45^

The beneficial effects of hearing aid use are also supported by trial evidence. Choi et al.^46^ found that use of a hearing aid was associated with a significant decrease in depressive symptoms at 6 months but not at 12 months, and that the most substantial improvements in depressive scores were seen in individuals who had the highest depressive symptoms at baseline. Our study suggests that the benefits of hearing aid use may extend beyond 12 months for mood symptoms. Recent randomized trial evidence further supports this, with significantly reduced depressive symptoms after 6 months in older adults offered over-the-counter hearing aids.^47^ Ongoing clinical trials are required to identify which groups may benefit most from hearing remediation.^48^

This study had several strengths and limitations. Limitations include bias due to attrition and nonresponse, a population that was free of dementia, independence-limiting physical disability, and known cardiovascular disease, which may limit generalizability. Furthermore, we classified depression using a screening tool (CES-D-10) rather than a clinician-established diagnosis. This scale assesses symptoms over the preceding week. Consequently, some cases may reflect transient affective states or context-appropriate distress rather than depressive disorder.^49^ Such misclassification is likely nondifferential with respect to hearing status and would bias associations toward the null, although differential reporting cannot be fully excluded. In addition, antidepressant treatment during follow-up may have influenced observed symptom trajectories. To reduce inclusion of prevalent treated depression, our first-onset analyses excluded participants with baseline CES-D-10 ≥ 8 and those using antidepressants at baseline. We did not adjust for post-baseline antidepressant use in Cox models because initiation typically follows depressive symptom emergence and thus functions as a time-varying mediator. Conditioning on this mediator would bias estimates of the total association between time-updated hearing status and incident depression.

There were several notable strengths, including the large sample size, use of audiometric measures, use of time-updating measures, modeling depression as a recurrent event, and extended follow-up period. The results provide valuable longitudinal evidence that moderate or severe HL is associated with an increased risk of depression among older adults, and notably, that hearing aid adherence may protect against this risk. By differentiating between minimal and extensive hearing aid use, our findings suggest that sporadic hearing aid use may not yield the same mental health benefit as consistent daily use of 6 h or more.

Still, questions remain about the precise mechanisms through which hearing aids may alleviate depressive symptoms, whether through improved social engagement, greater self-efficacy, or impacting biological mechanisms. Hearing loss may heighten cognitive load and alter auditory-limbic connectivity, leading to structural brain changes that impair mood regulation.^50^ Alternatively, shared neurodegenerative or vascular processes may contribute to both hearing decline and depression.^3^

Future research, including larger randomized trials in diverse populations, is necessary to pinpoint how best to optimize hearing aid use, explore barriers to consistent wear, and evaluate targeted interventions for older adults at heightened risk of depression. Ultimately, our findings underscore the importance of assessing and addressing hearing impairment as part of comprehensive geriatric care, with the potential to bolster not just communication abilities but also emotional well-being and overall quality of life in later years. Future research should investigate whether the relationship between HL and late-life depression is bidirectional, as depression may also affect adherence and response to HL treatment.

## Conclusion

Moderate-severity hearing impairment is a significant risk factor for depression in older adults. Hearing aid use may reduce the incidence of late-life depression. This has important clinical implications and should lead to targeted interventions and prevention strategies.

## Data Availability

Data are available via request to the principal investigator.

## References

[1] World Health Organisation. Deafness and hearing loss. Internet. WHO. Accessed November 26, 2024. 2024. https://www.who.int/health-topics/hearing-loss

[2] Kiely KM , Anstey KJ, Luszcz MA. Dual sensory loss and depressive symptoms: the importance of hearing, daily functioning, and activity engagement. Front Hum Neurosci. 2013;7:837. 10.3389/fnhum.2013.00837 24379769 PMC3864127

[3] Rutherford BR , Brewster K, Golub JS, Kim AH, Roose SP. Sensation and psychiatry: linking age-related hearing loss to late-life depression and cognitive decline. Am J Psychiatry. 2018;175:215-224. 10.1176/appi.ajp.2017.17040423 29202654 PMC5849471

[4] Andrade C , Pereira CR, Silva P. The silent impact of hearing loss: using longitudinal data to explore the effects on depression and social activity restriction among older people. Ageing Soc. 2017;38:2468-2489. 10.1017/S0144686X17000708

[5] Amieva H , Ouvrard C, Meillon C, Rullier L, Dartigues JF. Death, depression, disability, and dementia associated with self-reported hearing problems: a 25-year study. J Gerontol A Biol Sci Med Sci. 2018;73:1383-1389. 10.1093/gerona/glx250 29304204

[6] Lawrence BJ , Jayakody DMP, Bennett RJ, Eikelboom RH, Gasson N, Friedland PL. Hearing Loss and depression in older adults: a systematic review and meta-analysis. Gerontologist. 2020;60:e137-e154. 10.1093/geront/gnz009 30835787

[7] Powell DS , Betz JF, Yaffe K, et al Hearing loss and risk of depressive symptoms in older adults in the Health ABC study. Front Epidemiol. 2022;2:980476. 10.3389/fepid.2022.980476 38455326 PMC10910912

[8] Han JH , Lee HJ, Jung J, Park EC. Effects of self-reported hearing or vision impairment on depressive symptoms: a population-based longitudinal study. Epidemiol Psychiatr Sci. 2019;28:343-355. 10.1017/S2045796018000045 29415786 PMC6998913

[9] Tsimpida D , Kontopantelis E, Ashcroft DM, Panagioti M. The dynamic relationship between hearing loss, quality of life, socioeconomic position and depression and the impact of hearing aids: answers from the English Longitudinal Study of Ageing (ELSA). Soc Psychiatry Psychiatr Epidemiol. 2022;57:353-362. 10.1007/s00127-021-02155-0 34383085 PMC8784360

[10] Cosh S , Carriere I, Daien V, et al; Sense-Cog Consortium. The relationship between hearing loss in older adults and depression over 12 years: findings from the Three-City prospective cohort study. Int J Geriatr Psychiatry. 2018;33:1654-1661. 10.1002/gps.4968 30209835

[11] Liu W , Yang C, Liu L, Kong G, Zhang L. Bidirectional associations of vision loss, hearing loss, and dual sensory loss with depressive symptoms among the middle-aged and older adults in China. J Affect Disord. 2022;301:225-232. 10.1016/j.jad.2022.01.066 35038482

[12] Liu YG , Wang CC, Huang Q, Zhang L, Liu Y. Association of vision and hearing status with depressive symptoms among middle-aged and older Chinese adults. Front Public Health. 2022;10:857307. 10.3389/fpubh.2022.857307 35979465 PMC9376298

[13] Lisan Q , van Sloten TT, Lemogne C, et al Association of hearing impairment with incident depressive symptoms: a community-based prospective study. Am J Med. 2019;132:1441-1449.e4. 10.1016/j.amjmed.2019.05.039 31247178

[14] Kim JY , Lee JW, Kim M, Kim MJ, Kim DK. Association of idiopathic sudden sensorineural hearing loss with affective disorders. JAMA Otolaryngol Head Neck Surg. 2018;144:614-621. 10.1001/jamaoto.2018.0658 29852049 PMC6145778

[15] Kim HJ , Jeong S, Roh KJ, Oh YH, Suh MJ. Association between hearing impairment and incident depression: a nationwide follow-up study. Laryngoscope. 2023;133:3144-3151. 10.1002/lary.30654 36896880

[16] Hsu WT , Hsu CC, Wen MH, et al Increased risk of depression in patients with acquired sensory hearing loss: a 12-year follow-up study. Medicine (Baltimore). 2016;95:e5312. 10.1097/MD.0000000000005312 27858911 PMC5591159

[17] Simning A , Fox ML, Barnett SL, Sorensen S, Conwell Y. Depressive and anxiety symptoms in older adults with auditory, vision, and dual sensory impairment. J Aging Health. 2019;31:1353-1375. 10.1177/0898264318781123 29896982 PMC6274614

[18] Brewster KK , Ciarleglio A, Brown PJ, et al Age-related hearing loss and its association with depression in later life. Am J Geriatr Psychiatry. 2018;26:788-796. 10.1016/j.jagp.2018.04.003 29752060 PMC6008216

[19] Boorsma M , Joling K, Dussel M, et al The incidence of depression and its risk factors in Dutch nursing homes and residential care homes. Am J Geriatr Psychiatry. 2012;20:932-942. 10.1097/JGP.0b013e31825d08ac 22828203

[20] Chou KL. Combined effect of vision and hearing impairment on depression in older adults: evidence from the English Longitudinal Study of Ageing. J Affect Disord. 2008;106:191-196. 10.1016/j.jad.2007.05.028 17602753

[21] Cosh S , von Hanno T, Helmer C, Bertelsen G, Delcourt C, Schirmer H; SENSE-Cog Group. The association amongst visual, hearing, and dual sensory loss with depression and anxiety over 6 years: The Tromsø Study. Int J Geriatr Psychiatry. 2018;33:598-605. 10.1002/gps.4827 29193338

[22] Prince MJ , Harwood RH, Thomas A, Mann AH. A prospective population-based cohort study of the effects of disablement and social milieu on the onset and maintenance of late-life depression. The Gospel Oak Project VII. Psychol Med. 1998;28:337-350. 10.1017/s0033291797006478 9572091

[23] Pronk M , Deeg DJ, Smits C, et al Prospective effects of hearing status on loneliness and depression in older persons: identification of subgroups. Int J Audiol. 2011;50:887-896. 10.3109/14992027.2011.599871 21929374

[24] Lin CS , Lin YS, Liu CF, Weng SF, Lin C, Lin BS. Increased risk of sudden sensorineural hearing loss in patients with depressive disorders: population-based cohort study. J Laryngol Otol. 2016;130:42-49. 10.1017/S0022215115002960 26611115

[25] Killeen OJ , Xiang X, Powell D, et al Longitudinal associations of self-reported visual, hearing, and dual sensory difficulties with symptoms of depression among older adults in the United States. Front Neurosci. 2022;16:786244. 10.3389/fnins.2022.786244 35153667 PMC8829390

[26] Li X , Liu L, Luo N, et al Association of changes in self-reported vision and hearing impairments with depressive symptoms in middle-aged and older adults: evidence from a nationwide longitudinal study in China. Arch Gerontol Geriatr. 2024;116:105131. 10.1016/j.archger.2023.105131 37552924

[27] Xie T , Liu D, Guo J, Zhang B. The longitudinal effect of sensory loss on depression among Chinese older adults. J Affect Disord. 2021;283:216-222. 10.1016/j.jad.2021.01.081 33561802

[28] Sakurai R , Kawai H, Suzuki H, et al Cognitive, physical, and mental profiles of older adults with misplaced self-evaluation of hearing loss. Arch Gerontol Geriatr. 2023;104:104821. 10.1016/j.archger.2022.104821 36116286

[29] Triolo F , Belvederi Murri M, Calderón-Larrañaga A, et al Bridging late-life depression and chronic somatic diseases: a network analysis. Transl Psychiatry. 2021;11:557. 10.1038/s41398-021-01686-z 34718326 PMC8557204

[30] Jafari Z , Kolb BE, Mohajerani MH. Age-related hearing loss and tinnitus, dementia risk, and auditory amplification outcomes. Ageing Res Rev. 2019;56:100963. 10.1016/j.arr.2019.100963 31557539

[31] Cuda D , Manrique M, Ramos Á, et al Improving quality of life in the elderly: hearing loss treatment with cochlear implants. BMC Geriatr. 2024;24:16. 10.1186/s12877-023-04642-2 38178036 PMC10768457

[32] Ferguson MA , Kitterick PT, Chong LY, Edmondson-Jones M, Barker F, Hoare DJ. Hearing aids for mild to moderate hearing loss in adults. Cochrane Database Syst Rev. 2017;9:CD012023. 10.1002/14651858.CD012023.pub2 28944461 PMC6483809

[33] Cantuaria ML , Pedersen ER, Waldorff FB, et al Hearing loss, hearing aid use, and risk of dementia in older adults. JAMA Otolaryngol Head Neck Surg. 2024;150:157-164. 10.1001/jamaoto.2023.3509 38175662 PMC10767640

[34] McNeil JJ , Woods RL, Nelson MR, et al; ASPREE Investigator Group. Baseline characteristics of participants in the ASPREE (ASPirin in Reducing Events in the Elderly) study. J Gerontol A Biol Sci Med Sci. 2017;72:1586-1593. 10.1093/gerona/glw342 28329340 PMC5861878

[35] McNeil JJ , Woods RL, Ward SA, et al Cohort profile: the ASPREE Longitudinal Study of Older Persons (ALSOP). Int J Epidemiol. 2019;48:1048-1049h. 10.1093/ije/dyy279 30624660 PMC6693806

[36] Lowthian JA , Britt CJ, Rance G, et al; ASPREE Investigators. Slowing the progression of age-related hearing loss: rationale and study design of the ASPIRIN in HEARING, retinal vessels imaging and neurocognition in older generations (ASPREE-HEARING) trial. Contemp Clin Trials. 2016;46:60-66. 10.1016/j.cct.2015.11.014 26611434 PMC6753783

[37] Olusanya BO , Davis AC, Hoffman HJ. Hearing loss grades and the International classification of functioning, disability and health. Bull World Health Organ. 2019;97:725-728. 10.2471/blt.19.230367 31656340 PMC6796665

[38] Britt CJ , Storey E, Woods RL, et al; ASPREE Investigators. Age-related hearing loss: a cross-sectional study of healthy older Australians. Gerontology. 2024;71:1-12. 10.1159/000541895 PMC11854973 39571551

[39] Andresen EM , Malmgren JA, Carter WB, Patrick DL. Screening for depression in well older adults: evaluation of a short form of the CES-D. Am J Prev Med. 1994;10:77-84. 10.1016/S0749-3797(18)30622-6 8037935

[40] Wei J , Li Y, Gui X. Association of hearing loss and risk of depression: a systematic review and meta-analysis. Front Neurol. 2024;15:1446262. 10.3389/fneur.2024.1446262 39497727 PMC11532142

[41] R Core Team. R: A Language and Environment for Statistical Computing. R Foundation for Statistical Computing; 2024.

[42] Gopinath B , Schneider J, Hickson L, et al Hearing handicap, rather than measured hearing impairment, predicts poorer quality of life over 10 years in older adults. Maturitas. 2012;72:146-151. 10.1016/j.maturitas.2012.03.010 22521684

[43] Kamil RJ , Genther DJ, Lin FR. Factors associated with the accuracy of subjective assessments of hearing impairment. Ear Hear. 2015;36:164-167. 10.1097/aud.0000000000000075 25158982 PMC4272625

[44] Zhang ZQ , Li JY, Ge ST, et al Bidirectional associations between sensorineural hearing loss and depression and anxiety: a meta-analysis. Front Public Health. 2023;11:1281689. 10.3389/fpubh.2023.1281689 38259802 PMC10800407

[45] Salonen J , Johansson R, Karjalainen S, Vahlberg T, Jero JP, Isoaho R. Hearing aid compliance in the elderly. B-ENT. 2013;9:23-28. 23641587

[46] Choi JS , Betz J, Li L, et al Association of using hearing aids or cochlear implants with changes in depressive symptoms in older adults. JAMA Otolaryngol Head Neck Surg. 2016;142:652-657. 10.1001/jamaoto.2016.0700 27258813 PMC12861333

[47] Jiang F , Dayimu A, Dong Q, et al Over-the-counter hearing aids reduce depression symptoms in older adults with hearing loss: a randomized controlled trial. Nat Mental Health. 2025;3:498-506. 10.1038/s44220-025-00408-4

[48] Brewster KK , Pavlicova M, Stein A, et al A pilot randomized controlled trial of hearing aids to improve mood and cognition in older adults. Int J Geriatr Psychiatry. 2020;35:842-850. 10.1002/gps.5311 32291802 PMC7656495

[49] Horwitz AV , Wakefield JC, The Loss of Sadness: How Psychiatry Transformed Normal Sorrow into Depressive Disorder. Oxford University Press; 2007. 10.1176/appi.ajp.2007.07081263 22688233

[50] Griffiths TD , Lad M, Kumar S, et al How can hearing loss cause dementia? Neuron. 2020;108:401-412. 10.1016/j.neuron.2020.08.003 32871106 PMC7664986

